# 

**Published:** 1920-05-22

**Authors:** 


### Lord Riddell's Appeal.

Lord Riddell is presiding at the fifty-ninth Festival Dinner in aid of the funds of the Royal Hospital and Home for Incurables, Putney, which will be held on June 10 at the Savoy Hotel at 7 o'clock. He will be grateful for contributions however small, which should be sent to him at 8 Bouverie Street, Fleet Street, E.C. 4.

### St. Thomas's Hospital.

HOUSE APPOINTMENTS.—The following gentlemen have been selected as house officers from Tuesdays, May 4 and 18, 1920 (subject to the approval of the Treasurer):—

*Casualty Officers and Resident Anæsthetists.*—C. M. Billington, B.A., M.B., B.Ch. Cantab., M.R.C.S., L.R.C.P. (Ext.); J. Hale, M.A. Cantab., M.R.C.S., L.R.C.P. (Ext.); D. B. Spence, M.R.C.S., L.R.C.P. (Ext.); F. G. Wood, M.A. Cantab., M.R.C.S., L.R.C.P. (Ext.); D. G. Churcher, M.R.C.S., L.R.C.P.; R. M. Humphreys, B.A., M.B., B.Ch. Oxon.; R. C. P. Whitcombe, B.A. Cantab., M.R.C.S., L.R.C.P.; W. G. Woolrich, B.A. Cantab., M.R.C.S., L.R.C.P. *Resident House Physicians.*—W. S. Dawson, M.A., M.B., B.Ch. Oxon., M.R.C.S., L.R.C.P. (Ext.); P. C. Brett, M.R.C.S., L.R.C.P; S. A. T. Ware, M.R.C.S., L.R.C.P. (Ext.); M. L. Young, B.A., M.B., B.Ch. Cantab., M.R.C.S., L.R.C.P. (Ext.). *Resident House Physician (for Children).*—J. Forest Smith, M.R.C.S., L.R.C.P. (Ext.). *Resident House Surgeons.*—R. H. O. B. Robinson, B.A., M.B., B.Ch. Cantab., M.R.C.S., L.R.C.P. (Ext.); A. H. J. Smart, B.A., M.B., B.Ch. Cantab, M.R.C.S., L.R.C.P.; G. Perkins, M.C., M.A., M.B., B.Ch. Oxon., M.R.C.S., L.R.C.P. (Ext.); E. P. Brockman, B.A. Cantab., M.R.C.S., L.R.C.P. *Resident House Surgeon (for Ear, Nose, and Throat).*—H. I. Marriner, M.R.C.S., L.R.C.P. (Ext.). *House Surgeon to Block 8.*—L. G. Higgins, M.A., M.B. Cantab., M.R.C.S., L.R.C.P. (Ext.). *Obstetric House Physicians.*—H. L. Garson, O.B.E., M.C., B.A. Cantab., M.R.C.S., L.R.C.P.; R. C. Cooke, D.S.O., M.C., M.R.C.S., L.R.C.P *Ophthalmic House Surgeons.*—J. D. M. Cardell, M.R.C.S., L.R.C.P.; H. W. H. Holmes, B.A. Cantab., M.R.C.S., L.R.C.P. *Clinical Assistants.*—(Throat) J. B. Cavenagh, M.C., B.A., M.B., B.Ch. Oxon., M.R.C.S., L.R.C.P. (Ext.); C. H. C. Byrne, M.B., B.S. Lond., M.R.C.S., L.R.C.P. (Skin) W. A. Low, M.C., M.R.C.S., L.R.C.P.; A. C. Halliwell, B.A. Cantab., M.R.C.S., L.R.C.P. (Ext.). (Ear) J. B. Cavenagh, M.C., B.A., M.B., B.Ch. Oxon., M.R.C.S., L.R.C.P. (Ext.); C. H. C. Byrne, M.B., B.S. Lond., M.R.C.S., L.R.C.P. (Ext.). (Children's Medical) W. A. Low, M.C., M.R.C.S., L.R.C.P. (Electrical and *x*-ray Department) F. G. Spear, B.A. Cantab., M.R.C.S., L.R.C.P.; H. S. Le Marquand, M.R.C.S., L.R.C.P.; E. E. Carter, M.R.C.S., L.R.C.P. (Tuberculosis Department) F. B. Hobbs, B.A. Cantab., M.R.C.S., L.R.C.P.; A. C. V. Gossett, B.A. Cantab., M.R.C.S., L.R.C.P.

### Gifts and Bequests.

Mr. David Howell, J.P., of Cwmcynfelin, Cardiganshire, left £100 to Aberystwyth Infirmary.

Mr. Roger Beck, of Swansea, has purchased a local mansion, at a cost of £16,000, in order to give it to the public as a hospital annexe.

Mr. Robert Rurns, of Southport, has left £1,000 to St. Mary's Hospital, Manchester, and £1,000 to the Manchester Children's Hospital, Pendlebury.

Miss Margaret Hodgson, of Durham, has left £500 each to the Royal Victoria Infirmary, Newcastle-on-Tyne, the Darlington Hospital, and the Durham County Hospital.

Mr. Patrick Anderson Williamson, of Hoylake, Cheshire, bequeathed £1,000 to the Birkenhead Borough Hospital and £250 to the Birkenhead and Wirral Children's Hospital.

Mr. C. E. Groves, for some years Lecturer in Chemistry at Guy's Hospital, has left a reversionary legacy of £10,000 to the Royal Institution, Albemarle Street, W., for the "Groves Endowment Fund" for the promotion of scientific research.

Under the will of the late Viscount Portman £1,000 is left to Blandford County Hospital and £500 each to the Metropolitan Convalescent Institution, the Herbert Convalescent Institution, the Herbert Convalescent Home, Bournemouth, and the Samaritan Free Hospital.

Falkirk Infirmary is bequeathed a legacy of £1,000 under the will of Dr. John Aitken, LL.D., F.R.S.

Mr. John Wycherley, of 28 Hall Street, Southport, chartered accountant, left £1,000 to St. Dunstan's Hostel for the Blind.

Mr. Ewen Harper, F.R.I.B.A., architect of several public buildings in Birmingham, has left £200 each to the General Hospital, the Queen's Hospital, and the Children's Hospital.

Mr. Francis Skey, of Maida Vale, has left £2,000 each to St. Bartholomew's Hospital and the Prince of Wales's Hospital Fund, and £1,000 to the Essex County Hospital.

The Chelsea Hospital for Women has received a grant of £50 from the Worshipful Company of Clothworkers and £10 from St. Peter's (Cranley Guardians) Relief Committee.

Miss Ann Dawber, of Brookland Villa, Parbold, gave the income of her property for life to her sister, and on her sister's death to the Royal Albert Edward Infirmary, Wigan. The residuary bequest is believed to amount to about £10,000.

Mr. William Cann, a retired potato merchant, of Manchester, left £250 each to the Manchester Royal Infirmary, the Hospital for Incurables, Heaton Mersey, and St. Mary's Maternity Hospital, and £500 to the Orthopædic Hospital, Manchester.

### Forthcoming Events.

OLD LONDON AND THE LONDON HOSPITALS.—Under the auspices of the League of Mercy a lecture, illustrated by lantern slides, will be given on the above by Mr. G. Q. Roberts, C.B.E., on FRIDAY, MAY 28, at 5.15 P.M., at St. Thomas's Hospital (main central entrance). Admission free. Application for reserved seats to be made as early as possible to the Secretary, League of Mercy, 29 Southampton Street, Strand, W.C.

MISS ELLEN SYLK, M.B., M.R.C.S., has been appointed house surgeon at the Great Northern Central Hospital, Holloway.

### Matrons' and Sisters' Appointments.

COUNTY BOROUGH OF STOKE-ON-TRENT.—Miss Elizabeth Kelso Scott has been appointed matron-superintendent of the Certified Institution. She was trained at the Royal Albert Edward Infirmary, Wigan, and by the Queen Victoria's Jubilee Institute for Nurses, Scotland, has been head nurse, deputy matron, and later matron of the Midland Counties Institution, Knowle, assistant matron at the Cartcosh Hospital near Glasgow, and matron of Craig House, Royal Edinburgh Asylum.

NORTH LONSDALE HOSPITAL, BARROW-IN-FURNESS.—Miss Dora Comerford has been appointed out-patient sister. She was trained at the Royal Southern Hospital, Liverpool, and has been *x*-ray and orthopædic sister at Nelson Hospital, Merton. Miss Comerford holds the certificate of the Incorporated Society of Trained Masseuses, and also electricity certificates.

BATLEY AND DISTRICT HOSPITAL.—Miss Helen Gardner has been appointed matron. She was trained at Ancoats Hospital, Manchester, where she was later sister, theatre sister, and assistant matron. She has also held the post of home and theatre sister at Victoria Hospital, Blackpool, and is at present matron of Malton and District Hospital.

BELFAST INFIRMARY AND FEVER HOSPITAL.—Miss Mary E. Campbell has been appointed superintendent nurse. She was trained at the above institution, and has been assistant superintendent of the convalescent department, and for four and a-half years acting assistant superintendent of the Infirmary.

BIRMINGHAM MATERNITY HOSPITAL.—Miss Edith A. Meikle has been appointed matron. She was trained at the Royal Alexandra Infirmary, Paisley, where she was later staff nurse and ward sister. Miss Meikle has also held the posts of maternity ward sister at St. Mary's Hospital, Wakefield, night superintendent at the Clayton Hospital, Wakefield, maternity sister, and later assistant matron at the Nightingale Home, Derby.

FAVERSHAM INFIRMARY.—Miss M. O. Hobbs has been appointed sister. She was trained at St. James's Infirmary, Balham, and has served as sister under the British Red Cross Society.

STEPPING HILL HOSPITAL, STOCKPORT.—Miss Cicely Williamson has been appointed maternity sister. She was trained at the above-named institution, has held the posts of charge nurse, and senior charge nurse at the Basford Infirmary, Nottingham, and has done private nursing. Miss Williamson holds the Central Midwives Board certificate, and is a member of the College of Nursing.

WAKEFIELD UNION INFIRMARY.—Miss Margaret Fairhurst has been appointed assistant superintendent nurse. She was trained at Manchester Union Infirmary, and has since been head nurse at Northwich Union Infirmary and sister at the Chester War Hospital and the Albert Infirmary, Winsford, Cheshire.

MANSFIELD DISTRICT HOSPITAL.—Miss Catherine M. C. Wade has been appointed sister of the men's accident ward. She was trained at Bolton Infirmary and at the City Fever Hospital, Wakefield, and has since held the post of night sister at Macclesfield, and has been sister of the men's surgical ward at Huddersfield Royal Infirmary. Miss Wade is a member of the College of Nursing.

ST. BARTHOLOMEW'S HOSPITAL, ROCHESTER.—Miss H. F. Wells and Miss Ethel Turner have been appointed sisters of the men's surgical ward and children's ward respectively. Miss Wells was trained at St. Bartholomew's Hospital, London, and has been sister of the children's ward at St. Bartholomew's Hospital, Rochester. Miss Turner was trained at Leicester Royal Infirmary.

COTTAGE HOSPITAL, MAIDENHEAD.—Miss Mabel Ensor has been appointed matron. She was trained at St. Thomas's Hospital, where she was later sister of a male medical ward. She has since been sister-in-charge of a ward at No. 5 London General Hospital, matron in charge of a unit in East Africa, and sister-in-charge of a block at St. Thomas's. Miss Ensor has been Mentioned in Despatches, and was awarded the Royal Red Cross, First Class.

